# Differential detection of alternatively spliced variants of Ciz1 in normal and cancer cells using a custom exon-junction microarray

**DOI:** 10.1186/1471-2407-10-482

**Published:** 2010-09-10

**Authors:** Faisal A Rahman, Naveed Aziz, Dawn Coverley

**Affiliations:** 1 Department of Biology, University of York, UK; 2 Parkwood Hospital, Blackpool, UK

## Abstract

**Background:**

Ciz1 promotes initiation of mammalian DNA replication and is present within nuclear matrix associated DNA replication factories. Depletion of Ciz1 from normal and cancer cells restrains entry to S phase and inhibits cell proliferation. Several alternative splicing events with putative functional consequences have been identified and reported, but many more variants are predicted to exist based on publicly available mRNAs and expressed sequence tags.

**Methods:**

Here we report the development and validation of a custom exon and exon-junction microarray focused on the human CIZ1 gene, capable of reproducible detection of differential splice-variant expression.

**Results:**

Using a pair of paediatric cancer cell lines and a pool of eight normal lines as reference, the array identified expected and novel CIZ1 splicing events. One novel variant (delta 8-12) that encodes a predicted protein lacking key functional sites, was validated by quantitative RT-PCR and found to be over-represented in a range of other cancer cell lines, and over half of a panel of primary lung tumours.

**Conclusions:**

Expression of CIZ1 delta 8-12 appears to be restricted to cancer cells, and may therefore be a useful novel biomarker

## Background

Ciz1 (Cip1 interacting zinc-finger protein 1) is a nuclear protein that appears to be encoded by 17 exons that map to chromosome 9q34 in man (Unigene Hs.212395). Ciz1 is expressed in a wide range of tissues [1] and contains N-terminal polyglutamine repeats, 3 zinc finger motifs, one matrin 3 domain at the C-terminal end, 2 putative nuclear localization signals and a number of cyclin-dependent kinase phosphorylation sites and cyclin-binding motifs [2]. Ciz1 was first identified through its interaction with p21/Cip1 [3], a cyclin-dependent kinase (CDK) inhibitor involved in regulation of the cell cycle and in cellular differentiation. We identified Ciz1 in a cell-free system that reconstitutes initiation of DNA replication, under the regulation of cyclin A/CDK2 [4]. Using cell-free and cell-based approaches we showed that Ciz1 stimulates initiation of DNA replication in late G1 phase nuclei [5] and that it cooperate with cyclin E and A in order to execute its function in DNA replication [2]. Further detailed analysis revealed that DNA replication activity resides in the N-terminal half of Ciz1, while the protein becomes tethered to non-chromatin nuclear matrix structures through C-terminal sequences that play a role in its localization to DNA replication factories [6]. Taken together the data suggest that Ciz1 plays a linker role between the DNA replication machinery and the sub-nuclear structures that organize their function.

CIZ1 is a hormone-responsive gene [7,8] and is thought to play a role in the development or progression of oestrogen responsive tumours. It has also been linked with certain paediatric cancers [1,9], where alternatively spliced transcripts are prevalent. Similarly, inappropriate expression of alternatively spliced transcript has been reported to occur in brain tissue from Alzheimer patients [10]. Previously, we surveyed over 600 CIZ1 expressed sequence tags that map to the CIZ1 Unigene cluster [9], to overview alternative splicing during development and disease. This highlighted three exons in the 5' end of Ciz1 that are commonly alternatively spliced and potentially mis-spliced under some circumstances, and suggested a range of other rarer splicing events.

In this study our aim is to look at CIZ1 variant expression in order to validate previously reported alternative splicing (AS) events, and to discover new ones. We chose to use an exon junction array because commonly used methods for splicing analysis such as RT-PCR, cDNA primer extension and nuclease protection mapping are extremely labour intensive for highly spliced genes, particularly when analysis of a large number of samples is expected. Therefore, we have developed an exon junction-array focused on the human CIZ1 gene that is capable of detecting all known variants and hypothetical variants of CIZ1. We present results generated with this exon array-tool, initially using a pair of paediatric cancer cell lines. This identified several novel AS events including one cancer-restricted novel variant that we chose to validate further. The functional implications of expression of this variant (which has partial deletion of *exon 8 *and *exon 12 *and skipping of *exon 9, 10 *and *11*) are discussed and potential applications highlighted.

## Methods

### Array and probe design

**Figure 1:**
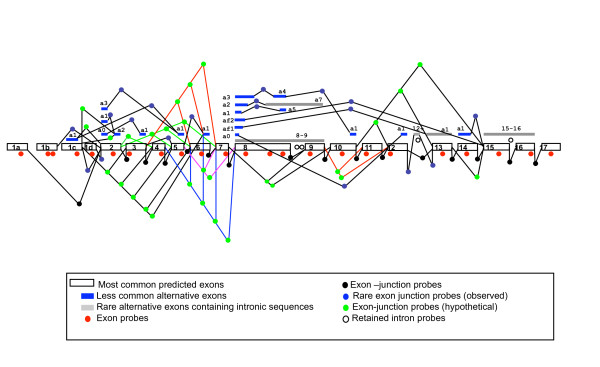
**Schematic representation of the location of human CIZ1 probes, designed to detect expression of the most common predicted exons, less common alternative exons, rare alternative exons containing intronic sequences, most common and observed rare exon-junctions and a selection of hypothetical junctions**. For junction probes between most common predicted exon, - indicates the junction such as Ciz1ex2-ex3. For junction probes between less common predicted exons, such as Ciz1 exon 2 alternative 1 and exon 2 alternative 2, the probe designation will be defined as Ciz1-ex2a1-ex2a2. Probe sequences are given in additional file 1 Table S1.

We designed oligonucleotide probes with extensive coverage for CIZ1 mRNAs and predicted variants. These include probes homologous to exons, observed junctions, hypothetical junctions and retained intron sequences (Fig. 1). The array also includes probes homologous to other genes that either emerged from our biochemical studies as Ciz1 interacting partners, have roles in cell cycle predicted to interact with Ciz1 or are known to be involved in pathology of sporadic cancers (additional file 1 Table S1). For 5' untranslated sequences we included probes that cover possible alternative first exons. These are defined as *exon 1a *(in NCBI Reference Sequence: NM_012127.2 and NM_001131015.1) and *exon 1c *(NM_001131017.1, NM_001131018.1 and NM_001131016.1). Other alternatively used exon 1 s were predicted by AceView (additional file 2 Figure S1) and confirmed by us when searching CIZ1 EST transcript against the BLAT CIZ1 assembly [11]http://genome.ucsc.edu/, and designated *exon 1b *and *1d *(additional file 3 Figure S2). To our knowledge inclusion of *exon 1b *and *1d *has not been verified by inclusion in a full-length human CIZ1 transcript.

To allow for a reasonable trade off between signal intensity and specificity, we restricted length of the probes to 40 nucleotides [12]. To increase specificity of probes, interspersed repeats and low complexity DNA sequences were masked (repeats have been replaced by Ns) using repeat masker [13]http://www.repeatmasker.org. The masked sequences were then searched for similarity against BLAT and areas which showed significant similarity were excluded. For junction probes, we employed the sliding window method where the 40 nt can slide across the target junction to allow for near similarity in melting temperatures [12]. Probes were then searched for similarity using BLAST non-redundant database with expectation value of 10 http://blast.ncbi.nlm.nih.gov/Blast.cgi[14]. Probes with more than 20 contiguous identical bases to non-self transcript were excluded. For all probes, exclusion criteria were set to include those with hairpin structures with stem exceeding 9 bases as well as those with self complementarities of more than 6 bases [15]. However, due to the constraints of the exon junctions, these parameters could not be applied to all probes. As the CIZ1 gene is rich in GCs, the target T_m _was set not to exceed 85°C (additional file 1, Table S1). Probes were synthesized by Operon Biotechnologies GmbH, Cologne, Germany. The oligonucleotide probes were printed on Nexterion Slides E (Schott) using a QArrayMini (Genetix) as follows: 5 replicates were printed for each probe in a random pattern including buffer as negative controls. Two slides from each batch of printing were analysed for spot quality using Ribogreen (Molecular Probes). Prior to hybridisation, each slide was washed at room temperature as follows: 1 × 5 min in 0.1% Triton X-100, 2 × 2 min in 1 mM HCL, 1 × 10 min in 100 mM KCL. Slides were subsequently blocked by incubation in 1 × Nexterion^® ^Blocking E (Schott) for 15 min and then dried by centrifugation. Microarray data associated with this paper was submitted to ArrayExpress (accession: E-MEXP-2331) with the experiment name 'Differential detection of alternatively spliced variants of Ciz1'.

### Cell culture and RNA extraction

A pool of eight stable cell lines of non-tumour origin were used as a reference control, (IMR90, WI38, HEK293, MRC5, HFL1, ASF-4-1, TIG-2M-30 and RPM1788, Table 1). Cell lines were obtained from the European Collection of Cell Cultures (ECACC) and the Japanese Collection of Research Bioresources (JCRB). Cells were cultured exactly as recommended. Total RNA was extracted using TRIZOL as recommended by the manufacturer (Invitrogen). RNA concentration was quantified using nanodrop spectrophotometer and equal concentrations from each of the eight samples were pooled together. SKNMC and TC466 Ewing tumour cell lines were used as test samples to validate the array and were handled as described previously [9].

**Table 1:** Cell lines used in this analysis

Cell line	Derivation	Reference
TTC466	Ewings Tumour	[23]

SKNMC	PNET	[24]

H727	Human bronchial carcinoid	[25]

IMR90	Human embryonic lung	[26]

WI38	Human embryonic lung	[27]

HEK293	Human embryonic kidney	[28]

MRC5	Human embryonic lung	[29]

HFL1	Human embryonic lung	[30]

ASF-4-1	Skin fibroblast from upper arm	[31]

RPMI 1788	normal blood, IgM secreting	[32]

TIG-2M-30	muscle, normal diploid fibroblast	[33]

### Preparation and labeling of templates

20 μg of pooled RNA reference control or test samples (SKNMC and TTC466) were reversed transcribed as described and labeled using Invitrogen protocol (SuperScript ™Plus Indirect cDNA labeling System). Labelled reference and test sample (20 μl each) were combined in an amber tube, mixed and heated at 99°C for three minutes before hybridization.

### Prehybridization and Hybridization

Prior to hybridization slides were prepared as mentioned previously. Hybridization was carried out in a final volume of 40 μl in Corning hybridization chambers, in a water bath at 42°C for 16-24 h. Microarray slides were then washed twice in 1× SSC with 0.1% SDS for five minutes, once in 0.1× SSC with 0.1% SDS for 5 minutes and a final wash in 0.05× SSC for five minutes. Slides were transferred to a centrifuge tray and spun for three minutes at 2000 rpm at room temperature to dry.

### Image acquisition and data analysis

Slides were scanned using GenePix 4000B microarray scanner and images were analyzed using GenePix software where a grid layout was applied to the features. Data analysis was performed using ArrayAssist software where background correction was done using foreground-background (FG-BG). Features with signal intensities less than 2.5 fold of the mean background signal were removed, and ratios were normalized using housekeeping gene normalization [16] and were averaged and log transformed.

### Reverse-Transcriptase PCR

cDNA was generated using superscript III Synthesis Kit according to manufacturer's instructions (Invitrogen). Normalization of input cDNA was done using Actin forward (5'CAACCGCGAGAAGATGACC3') and reverse primers (5'TCCAGGGCGACGTAGCA CA3') as endogenous control. For CIZ1 sequences spanning *exon 8 *to *exon 13*, primers Ciz1-ex8F (5'CTCCAGGGCAGTTACAGGAC3') and Ciz1-ex13R2 (5'TGCGAGGGGTT TTGAAGTAG3') were PCR amplified at 58°C annealing temperature using phusion high-fidelity DNA polymerase (Finnzymes). For the variant with partial deletion of *exon 8 *and *exon 12 *and skipping of *exon 9, 10 *and *11*, splice junction specific primers were used. Forward primer, Ciz1-jex8-ex12F (5'CTCCAGGGCAGTTACAGGAC3') and reverse primer hCiz1-Jex13-ex14R (5'CTCAAGCGACTTCAGCTCCT3') were PCR amplified at 60°C annealing temperature using Taq polymerase (New England Biolab (NEB). For alternative exon 1 s, forward primers Ciz1-ex1b (5'CAGACGGACCTTGGT CTCC3'), Ciz1-exon 1c (5'GCGACTTGAGCGTTGAGG3') and Ciz1-ex1d (5'GGCGG TGGTGGAGAGAAG3') were all combined with reverse primer Ciz1-ex5R (5'CGATTGG GGGTGGTAGAGG3') in separate reactions. Taq polymerase (NEB) was used for amplification at annealing temperatures of 60°C, 62°C and 56°C respectively.

### Quantitative Real-Time PCR

Reactions were carried out using an ABI 7000, initial incubation at 95°C/10 min, followed by 95°C/15 sec and 1 min at 60°C for 40 cycles. Triplicate reactions were carried out in 96-well plates in a 25 μl reaction volume containing 12.5 μl cyber green Master Mix (Applied Biosystems), 20 ng cDNA and 200 nM each of hCiz1-Jex8-ex12F forward and hCiz1-Jex13-ex14R reverse. Primers used for normalization include, Actin F and Actin R as well as GAPDH forward (5'ATCCCATCACCATCTTCCAGG3') and reverse (5'GCATC GCC CCACTTGATTTTG 3').

## Results and discussion

CIZ1 is represented by 865 GenBank entries that map to Unigene Build number 224, cluster Hs.212395. These mRNAs and ESTs were aligned cooperatively by AceView [17] to exclude redundancies producing 27 mRNA assemblies (additional file 2 Figure S1 1). These were then aligned to the gene sequence and exon boundaries and splice junctions identified. CIZ1 probes were designed from constitutive regions, alternative regions and observed exon-exon splice junctions. To allow for discovery of novel alternative splicing events, we also designed probes representing hypothetical exon-exon junctions. The array is designed to survey overall CIZ1 alternative splicing in different tissues, diseases and developmental stages, with the aim of substantiating observed alternative splicing features and identifying novel alternative splicing events. Here we discuss initial array results on the SKNMC PNET and TTC466 Ewing tumour cell lines.

**Figure 2:**
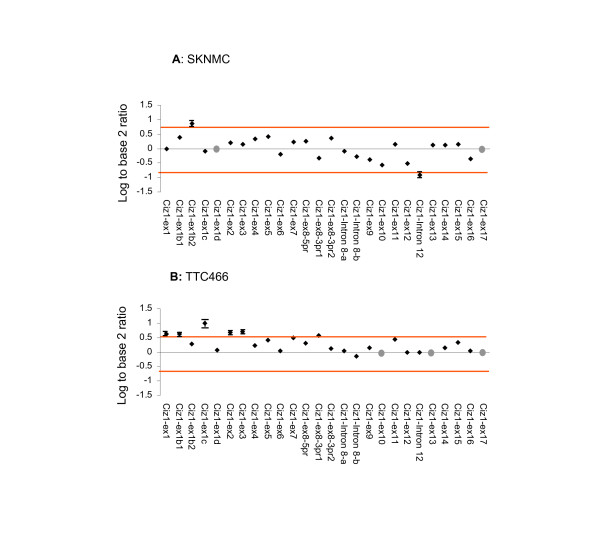
**Expression of CIZ1 in paediatric cancer cell lines revealed by hybridization to CIZ1 exon probes**. **A**: CIZ1 exon expression in primitive neural ectodermal tumour (PNET) cell line SKNMC. **B**: CIZ1 exon expression in Ewing tumour TTC466 cell line. Plots show the average of three technical replicates expressed as log^2 ^of the ratio between signal generated by the test sample and signal generated by reference RNA. Change is considered to be significant where this fall outside 0.58 or -0.58 and therefore exceed 1.5 fold relative to control. Bars indicating SEM are shown for probes included by these selection criteria. No reliable data was achieved for probes indicated with a grey dot.

**Figure 3:**
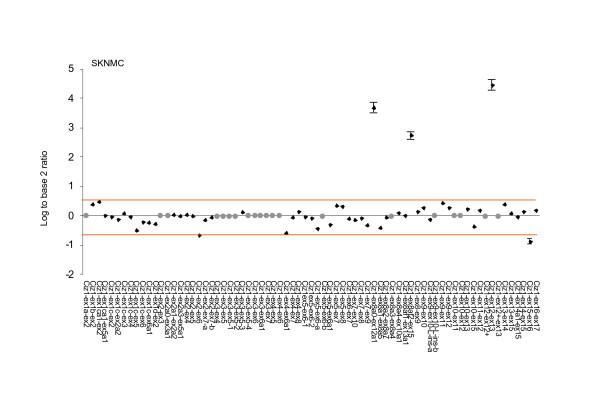
**Expression of CIZ1 in paediatric cancer cell lines revealed by hybridization to CIZ1 exon-junction probes for SKNMC PNET cell line**. Plots show the average of three technical replicates expressed as log^2 ^of the ratio between signal generated by the test sample and signal generated by reference RNA. Change is considered to be significant where this fall outside 0.58 or -0.58 and therefore exceed 1.5 fold relative to control. Bars indicating SEM are shown for probes included by these selection criteria. No reliable data was achieved for probes indicated with a grey dot.

**Figure 4:**
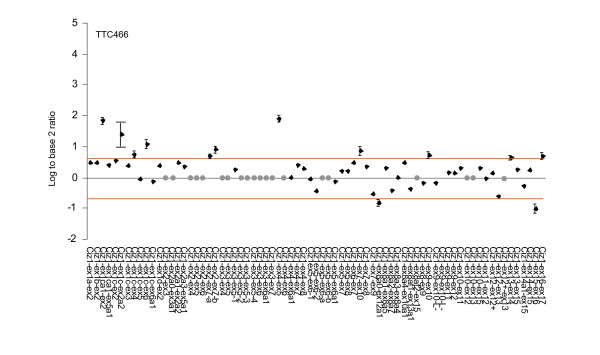
**Expression of CIZ1 in paediatric cancer cell lines revealed by hybridization to CIZ1 exon-junction probes for TTC466 Ewing tumour**. Plots show the average of three technical replicates expressed as log^2 ^of the ratio between signal generated by the test sample and signal generated by reference RNA. Change is considered to be significant where this fall outside 0.58 or -0.58 and therefore exceed 1.5 fold relative to control. Bars indicating SEM are shown for probes included by these selection criteria. No reliable data was achieved for probes indicated with a grey dot.

Analysis of the technical replicates in this study showed that out of 26 exon probes the data was consistent and informative for 24 probes (92%) for the SKNMC cell line and 23 probes (88.5%) for TTC466 cell line. Data was not obtained for the CIZ1 exon 17 probe in either cell line suggesting a problem with the probe. For the remaining 3 (Fig. 2A, B), their failure was cell-line specific, possibly reflecting severe under or over expression outside the detection parameters of this analysis. For the 73 CIZ1 junction probes used here reproducible data was not available for 5 junctions observed in the EST databases and 14 that were rare or hypothetical junctions for SKNMC cells (Fig. 3). Similarly for TTC466, of the 73 junction probes, data was not available for 4 observed junctions and 17 rare or hypothetical junctions (Fig. 4). Three of the observed and 11 of the rare or hypothetical junctions were common to both cell lines. We consider these figures to be within the expected range due to the constraints inherent in designing probes for exon junctions, coupled with the fact that some of the hypothetical junctions may never exist in reality. Of greater interest is the hypothetical junctions that were consistently represented in the transcriptome of these two cell lines. These are ex1ca1-ex2, ex1c-ex2a2, ex1c-ex4, ex1c-ex6, ex2-ex7 and ex7-ex10 (depicted graphically in additional file 3 Figure S2), indicating an unanticipated level of AS around untranslated *exons 1 *and *2*.

Analysis of 3 technical replicates for Ewings tumour cell line SKNMC showed that CIZ1 *exon 1b *is up-regulated and CIZ1 intron 12 (alternative exon) is down regulated relative to the reference template, although both fall only just within inclusion criteria of 1.5 fold change. When compared to 2 further biological replicates (isolated from separate population of SKNMC cells), neither probe proved to be consistently different to the reference template (Table 2 and Fig. 2A). However similar analysis of cell line TTC466 also indicated differential expression of the alternative *exon 1s*. In fact CIZ1 *exon 1c *was considerably up-regulated in all three biological replicates (Table 3), with an SEM of 0.355135 between the three technical replicates (Fig. 2B). Moreover among the exon 1c-junction probes, 4 were consistently up-regulated relative to the reference control in all 3 biological replicates and 3 of these were among the top 4 ranked products [18] (additional file 4 Table S2). This analysis revealed splicing of *exon 1c *onto *exon 2 *(variant 2), *exon 4 *and *exon 6 *and splicing of alternative *exon 1c *(1ca1) onto *exon 2 *(Table 3 and Fig. 3).

**Table 2:** Summary of CIZ1 alternative splicing in SKNMC cell line

SKNMC exon probes	Average of 3 technical replicates (B1)	Biological replicate 2	Biological replicate 3
Ciz1-ex1b2	+	=	=

Ciz1-intron 12	-	=	-

**SKNMC junction probes**			

Ciz1-ex8a0-ex12a1	+	+	+

Ciz1-ex8af2-ex15	+	+	=

Ciz1-ex12-ex13	+	+	+

Ciz1-ex15-ex16	-	=	=

Three independent RNA isolates from proliferating SKNMC cell (Biological replicates, B1, B2 and B3) were analysed on individual arrays, with one isolate undergoing analysis in triplicate (technical replicate, T1, T2 and T3 shown in Fig. 2A and 3). Probes that are up or down regulated more that 1.5 fold and who's SEM indicate agreement between three technical replicates are shown. Results for these probes derived from the two further biological replicates (replicate 2 and replicate 3) are also shown. + indicates significantly up-regulated, - indicates significantly down-regulated and = indicates no significant change. Significance is taken to be a change that is ≥ 1.5 fold compared to control RNA. For paediatric cancer cell line SKNMC, expression of two probes was consistently and significantly altered compared to control samples, these are Ciz1-ex8a0-ex12a1 and Ciz1-ex12-ex13. Raw data is given in Additional file 5, tables S4-S8.

**Table 3:** Summary of CIZ1 alternative splicing in TTC466 Ewing cell line

TTC466 exon probes	Average of 3 technical replicates (Biol. 1)	Biological replicate 2	Biological replicate 3
Ciz1-ex1	+	nd	=

Ciz1-ex1b1	+	=	=

Ciz1-ex1c	+	+	+

Ciz1-ex2	+	=	=

Ciz1-ex3	+	+	+

**TTC466 junction probes**			

Ciz1-ex1ca1-ex2	+	+	+

Ciz1-ex1c-ex2a2	+	+	+

Ciz1-ex1c-ex4	+	+	+

Ciz1-ex1c-ex6	+	+	+

Ciz1-ex2-ex7-a	+	+	+

Ciz1-ex2-ex7-b	+	=	=

Ciz1-ex4-ex5	+	+	+

Ciz1-ex7-ex10	+	+	+

Ciz1-ex8a0-ex12a1	-	nd	_

Ciz1-ex9-ex10	+	+	+

Ciz1-ex13-ex14	+	=	=

Ciz1-ex15-ex16	_	=	=

Ciz1-ex16-ex17	+	=	=

Three independent RNA isolates from proliferating TTC466 cells (Biological replicates, B1, B2 and B3) were analysed on individual arrays, with one isolate undergoing analysis in triplicate (technical replicate, T1, T2 and T3). Probes that are up or down regulated more that 1.5 fold and who's SEM indicate agreement between three technical replicates are shown. Results for these probes derived from the two further biological replicates (replicate 2 and replicate 3) are also shown. + indicates significantly up-regulated, - indicates significantly down-regulated and = indicate no significant change. For paediatric cancer cell line TTC466, expression of 2 exon probes was consistently and significantly altered compared to control samples, these are Ciz1-ex1c and Ciz1-ex3. For exon junction probes a total of 8 probes met all our selection criteria. These are Ciz1-ex1ca1-ex2, Ciz1-ex1c-ex2a2, Ciz1-ex1c-ex4, Ciz1-ex1c-ex6, Ciz1-ex2-ex7-a, Ciz1-ex4-ex5, Ciz1-ex7-ex10 and Ciz1-ex9-ex10. Raw data is given in Additional file 6, tables S9-S13.

Other genes in this study that were overrepresented in all technical and biological replicates were RB1 and VPS72 for SKNMCS cell line, and CDC2, CDC6, TNRC9 and DDX17 in TTC466 cells (additional files 6 and 7 Tables S4-S13). VPS72 is an interacting partner of EWSR1 gene [19]. RB1, CDC2 and CDC6 may share the same pathways as CIZ1 gene. Expression of these genes needs to be validated and their function with regard to Ciz1 further analysed.

### Expression of alternative exon 1s

We surveyed CIZ1 ESTs using BLAT [11] to review alternative usage of *exon 1*. This showed that CIZ1 *exon 1c *is supported by 4mRNAs and 139 ESTs which exceeds by far the number of mRNAs and ESTs supporting other exon 1 s. The second most common is *exon 1b *supported by 16 ESTs. This analysis might not reflect the true nature of *exon 1 *usage due to possible bias towards 3' exons. In this study, we detected over-expression of the novel alternative *exon 1b *in technical replicates of both SKNMC and TTC466 cell lines (Tables 2 and 3), however no change was observed in biological replicates of the two cell lines. On the other hand for *exon 1c*, array results showed consistent up-regulation in TTC466 cell line as well as the junctions of *exon 1c *and other exons (*2, 4 *and *6*). Taken together these results suggest that *exon 1c *is the most widely used alternative *exon 1 *for the CIZ1 gene. Protein translation for alternative usage of *exon 1 s *on the background of full length Ciz1 showed that *exon 1c *and *1d *would encode the same protein. On the other hand translation for *exon 1b *showed there are two additional predicted methionines upstream of the usual predicted methionine in *exon 2*, adding an extra 28 amino-acids to the N-terminal end of the Ciz Protein. 5' UTR selection has been shown to affect alternative splicing of downstream exons. For example, for oestrogen receptor β it has been reported that alternative 5' UTR influences splicing and may alter protein function [20]. Furthermore, inappropriate expression of 5' UTRs has been implicated in carcinogenesis in the case of BRCA1 [21] and Mdm2 [22]. The functional significance of CIZ1 alternative *exon 1 *expression requires further investigation, however their expression may play a role in specifying alternative splicing of the rest of the CIZ1 gene.

### Exclusion of exon 4

Previously, we showed that an alternatively spliced human CIZ1 variant, with deletion of *exon 4 *(ΔE4), is mis-expressed as a consequence of intronic mutation in Ewings Tumour (ET) cell lines, including the two cell lines used here [9]. However, skipping of e*xon 4 *was not reproducibly detected in either cell line. This is most likely due to constraint inherent in the sequence of CIZ1 junction exon 3-exon 5. Thus, this approach is not suitable for further investigation of alternative splicing at this site.

### A novel cancer-associated variant

In addition to extensive AS of *exon 1c *, for TTC4666 cells a further 9 probes were significantly under or over represented in 3 technical replicates compared to the reference control, and four of these (2-7a, 4-5, 7-10, 9-10) were reproducibly detected in 2 further biological replicates. We were most interested in one probe (8a0-12a1) that is down-regulated in 3 of 3 technical replicates and 2 of 3 biological replicates in TTC466 cells, because the same probe is up-regulated in all replicate experiments carried out with SKNMC cells. This is the only probe that is significantly altered in both cell lines, albeit upregulated in one and down regulated in the other. This probe indicates expression of a CIZ1 splice variant with partial deletion of *exon 8 *and *12 *and skipping of *exon 9, 10 *and *11*, and is the top one of 4 ranked products for SKNMC cells (additional file 4 Table S2).

**Figure 5:**
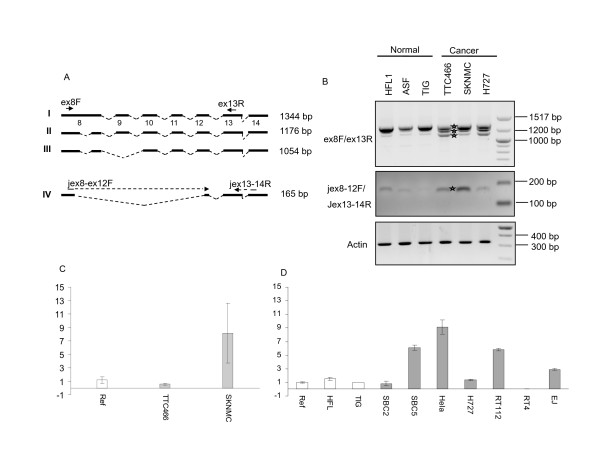
**Detection of over expressed CIZ1 spliced variants in cancer cell lines**. **A**: Schematic representation of CIZ1 *exon 8 *to *exon 13 *and the primers (Ciz1-ex8F-Ciz1-ex13R) used for amplification. Schematics **I-III **corresponds to the 3 bands seen in Fig. 5B (upper panel) which were verified by sequencing. Schematic **I **represents full length *exon 8-13*, **II **shows partial deletion of *exon 8 *and **III **shows partial deletion of *exon 8 *and skipping of *exon 9*. Schematic **IV **indicates primers used to amplify the product shown in Fig. 5B (middle panel) and in Fig. 5C and D. **B**: Upper, RT-PCR using the primer pair Ciz1-ex8F and Ciz1-ex13 R. Middle, RT-PCR using variant specific primers (Ciz1-jex8-ex12F - Ciz1-jex13-ex14R). Bottom, actin primers were used for normalization. The panel includes three normal cell lines (HFL1, ASF and TIG) and three cancer cell lines TTC466, SKNMC and H727). Stars indicate sequenced bands. **C**: Validation of microarray result for *exon 8-12 *with SKNMC and TTC466. Histogram shows average quantitative PCR result for 3 replicates with SEM. Results were normalised to Actin levels and calibrated to pooled reference RNA (8 normal cell lines including, IMR90, WI38, HEK293, MRC5, HFL1, ASF-4-1, TIG-2M-30 and RPM1788), as used in the microarray experiment. **D**: Quantitative PCR with Ciz1 splice variant specific primers (Ciz1-jex8-ex12F and Ciz1-jex13-ex14R). The panel includes lung (SBC2, SBC5 and H727), cervix (Hela), and Urothelial cancer cell lines (RT112, RT4 and EJ). Normal cell lines were also included (HFL and TIG) as well as the reference poole RNA, which was used as calibrator. Results were normalized to GAPDH.

**Figure 6:**
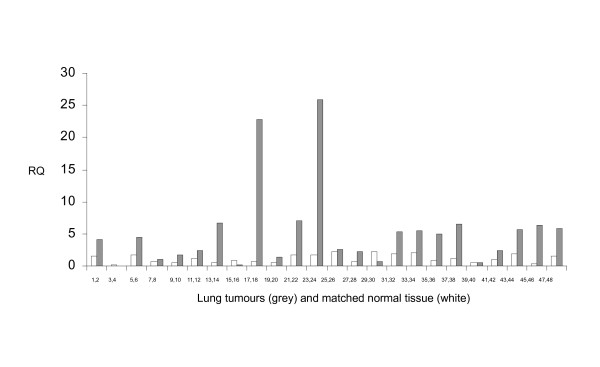
**Quantitative PCR with CIZ1 splice variant specific primers (Ciz1-jex8-ex12F and Ciz1-jex13-ex14R) in Lung cancer**. The lung cancer Tissue Scan cDNA array (HLRT504, Origene) contains matched pair sets of 48 tissues covering four disease stages and normal tissues. Each pair set is calibrated to its normal tissue and results were normalized to Actin. Grey columns indicates tumour and white columns indicates paired normal tissue.

To further investigate alternative splicing between *exon 8 *and *12 *in human CIZ1 (Fig. 5A), we used RT-PCR from exon 8 (forward primer 8F) coupled with a reverse primer in *exon 13 *(13R). Results showed 3 transcripts in Ewings tumour cell lines and also in a third cancer cell line H727 (human lung carcinoid). Three products of the same mobility were detectable in 3 normal cell lines, but at considerably lower levels for the smaller of the three (Fig. 5B, upper). Thus, these products are over-expressed in cancer cell lines. Sequencing revealed the upper band (1344bp) to be the expected CIZ1 full-length sequence (Reference sequence NM_0121276.2, Fig. 5A), the middle band to lack sequences internal to exon 8 (168 bp) and the lower band to lack this region plus the whole of *exon *9 (1054 bp). Taking NM_012127.2 as a reference sequence the *exon *9 deletion is described as c.1338_1505del168 and exon 9 skipping as r.1702_1823del. Despite its prevalence in the cancer cell lines we studied, to our knowledge, this is the first time this variant has been reported. Neither of these events is represented by probes in our array. Notably, the variant detected by our array that splices *exon *8 to *exon *12 (c1038_2218del1181) was barely detectable by this analysis. Therefore in order to quantify its expression we designed specific primers Ciz1-jex8-ex12F-Ciz1-jex13-ex14R (Fig. 5A). Results showed increased expression in SKNMC cells compared to 3 normal cell lines (HFL, ASF and TIG) and comparable expression in TTC466 cells (Fig. 5B, middle). The 165 bp product was sequenced, verified for SKNMC and TTC466 and found to be as expected. Similar results were obtained by quantitative real time PCR after normalisation to actin levels and calibration to the pooled reference RNA used in the arrays. As expected, results showed significant over expression for this variant (exon 8-exon 12) in SKNMC and slight down-regulation in TTC466 cell line (Fig. 5C), validating results obtained by array analysis. Quantitative detection of this apparently cancer-associated variant was extended to cover other cancer cell lines derived from lung (SBC2, SBC5 and H727), cervix (Hela) and urothelial cells (RT112, RT4 and EJ). Two individual normal cell lines HFL and TIG, as well as the pooled reference RNA were included as controls. Significant (greater than 2 fold) over- expression was detected in four of the 7 cancer cell lines (Fig. 5D). Furthermore, using Tissue Scan lung cancer array HLRT504 (Origene), we performed quantitative real-time PCR on cDNA obtained from 48 tissues. Results showed significantly elevated levels of this CIZ1 splice variant in 13 tumour samples compared to the matched paired normal controls (Fig. 6). Thus, the data indicate that exclusion of *exon 9, 10, 11 *and part of *exon *8 and *exon *12 is a recurrent event in cancer cells. This variant (c1038_2218del1181) lacks sequences that encode motifs that are important for the function of Ciz1 in DNA replication. Bioinformatic analysis revealed that the excluded sequence encodes as many as 6 putative CDK phosphorylation sites and 2 cyclin interaction sites (CY motifs). Notably sequences encoded by *exon *9 engage in sequential interaction with cyclin E and A, and support functional cooperation with cyclinA/CDK2 driving initiation of DNA replication *in vitro *[2]. This analysis was carried out with murine Ciz1 protein which is 64% homologous at the protein level to human Ciz1 in this region (*exon *8-12). As the human variant described here is lacking these sequences our extrapolation is that it will not be active in DNA replication, despite most likely retaining down-stream sequences that anchor Ciz1 to the nuclear matrix [6]. Although further functional analyses are needed to understand the impact that expression of this variant has on DNA replication, these data suggest it may interfere with the normal function of Ciz1.

## Conclusion

Expression of variant 8a0-12a1 that is restricted to cancer cells may offer a useful marker for their detection. Furthermore, because siRNA depletion of Ciz1 has been shown to delay S phase entry and restrain cell proliferation [5], selective inhibition of CIZ1 variants have potential to offer selective restraint of those cell that express them. Thus variant 8a0-12a1 has potential application as a selective therapeutic target in some lung cancer patients.

## Abbreviations

PNET: primitive neuroectodermal tumor; Ciz1: CDKN1A interacting zinc finger protein 1; RT-PCR: reverse transcriptase polymerase chain reaction; AS: alternative splicing; EST: expressed sequence tag; BLAST: Basic Alignment Search Tool; BLAT: Blast-Like Alignment Tool; cDNA: DNA complementary to RNA; NEB: New England Biolab; RB1: retinoblastoma 1; VPS72: vacuolar protein sorting 72 homolog (S. cerevisiae); CDC2: cell division cycle 2; CDC6: cell division cycle 6 homolog (S. cerevisiae); TNRC9: TOX high mobility group box family member 3; DDX17: DEAD (Asp-Glu-Ala-Asp) box polypeptide 17; UTR: Un-Translated Region; BRCA1: breast cancer 1; Mdm2: Murine double minute 2; SEM: Standard Error of the Mean; GAPDH: glyceraldehyde-3-phosphate dehydrogenase.

## Competing interests

DC is partly supported by and holds shares in University of York spin-out Cizzle Biotechnology. The CIZ1 splice variant array, and the delta 8-12 variant described in this paper were patent protected by the University of York on 30 July 2009.

## Authors' contributions

FAR participated in the design of the study, carried out molecular genetic studies, statistical analysis, data interpretation and drafted the manuscript. NA participated in the design of the study, statistical and data interpretation. DC conceived of the study, and participated in its design and coordination and helped to draft the manuscript. All authors read and approved the final manuscript.

## Pre-publication history

The pre-publication history for this paper can be accessed here:

http://www.biomedcentral.com/1471-2407/10/482/prepub

## Supplementary Material

Additional file 1 **Table S1**. Probe sequences. Click here for file

Additional file 2 **Figure S1**. Schematic representation of predicted human Ciz1 alternatively spliced transcripts assembled by AceView from 865 mRNA and ESTs submitted to GenBank [17]. Blue lines represent exons, black represent introns, yellow represent 5' or 3' untranslated regions and * represent non canonical exon-intron boundaries. Alternative transcript assemblies were designated, B, A, E, F, G, H, I, J, K, L, M, P, Q, R, S, V, W, Y, Z, 27, 28, 29, 30, 31 and 32 by AceView. Click here for file

Additional file 3 **Figure S2**. Observed and hypothetical exon-junctions generated by alternative splicing of Ciz1 alternative *exon 1 s *(ex 1a, ex 1b, ex 1c and ex 1d). Common exons are indicated by labelled boxes and less common alternative exons by solid black boxes. Observed and hypothetical junctions are indicated by black and grey broken lines respectively. Splicing events that are over represented in TTC466 are indicated by solid lines. Sequences at 3' end of alternative *exon 1 s *are given in additional file 7 Table S14. Click here for file

Additional file 4 **Table S2**. Ranked product, top 4 up-regulated probes in technical replicates of SKNMC. **Table S3 **Ranked product, top 4 up-regulated probes in technical replicates of TTC466. Click here for file

Additional file 5 **Table S4**. microarray result for SKNMC technical replicate 1 (T1). **Table S5 **microarray result for SKNMC technical replicate 2 (T2). **Table S6 **microarray result for SKNMC technical replicate 3 (T3). **Table S7 **microarray result for SKNMC biological replicate 2 (B2). **Table S8 **microarray result for SKNMC biological replicate 3 (B3). Click here for file

Additional file 6 **Table S9**. microarray result for TTC466 technical replicate 1 (T1). **Table S10 **microarray result for TTC466 technical replicate 2 (T2). **Table S11 **microarray result for TTC466 technical replicate 3 (T3). **Table S12 **microarray result for TTC466 biological replicate 2 (B2). **Table S13 **microarray result for TTC466 biological replicate 3 (B3). Click here for file

Additional file 7 **Table S14**. Sequences of alternative exon 1 (3' end). Click here for file
