# Supplementary material for: Differential detection of alternatively spliced variants of Ciz1 in normal and cancer cells using a custom exon-junction microarray
Source: BMC Cancer. 2010 Sep 10;10:482. doi: 10.1186/1471-2407-10-482 (PMC2945943; doi:10.1186/1471-2407-10-482)
Supplement: Additional file 1 — Table S1. Probe sequences. [file 1471-2407-10-482-S1.DOC]

Table S1: probe sequences

| probe | sequence | | TM |
| --- | --- | --- | --- |
| Probes for Ciz1 most common predicted exons | | |  |
| Ciz1 ex1a | GGCCGTGGCGAAGCAAGGGGCCGGCTGCTCAGAAAAGGAT | | 79.05 |
| Ciz1-ex2 | AGCTCCAGCAGCTGCTCCAGCAGTCCCCACCACAGGCCCC | | 81.1 |
| Ciz1-ex3 | GGGGCTCCCCCCGCAGCAGCCACAGCAGCCGCTTCTGAAT | | 81.1 |
| Ciz1-ex4 | TATGACACTGCCGGTCTCACCATGCCCACAGCAACACTGG | | 75.97 |
| Ciz1-ex5 | ACAGGCCCGGACCTCCTCCTCTACCACCCCCAATCGAAAG | | 78.03 |
| Ciz1-ex6 | CAGACAATGCCTGTGGAAGACAAGTCAGACCCCCCAGAGG | | 75.97 |
| Ciz1-ex7 | CCTTGTGAGGCGTCCGAGCTGCCAGCAAAGAGATTGAGGA | | 75.97 |
| Ciz1-ex8-5pr | CCGCAGGCCCGGATGACAGTACCGAAACAGACACAGACAC | | 77 |
| Ciz1-ex8-3pr1 | GGAGCATCCTCCAGCGCAGGTGTCAGTACAGCCACCAGAG | | 78.03 |
| Ciz1-ex8-3pr2 | CACCAGTTGTGGTTCATGTCTGCGGGCTGGAGATGCCACC | | 77 |
| Ciz1-ex9 | TGGGCACCCAAGTCAGCATGGAAGAGATTCAGAATGAGTC | | 72.9 |
| Ciz1-ex10 | CAGTTCTTCTGCTACATCTGCAAGGCCAGCTGCTCCAGCC | | 75.97 |
| Ciz1-ex11 | AGCCTCAGCACCAGCAGCGGCTAGGGGAGATCCAGCACAT | | 78.03 |
| Ciz1-ex12 | GCAACACCTGCCAGCTCTACTACATGGGGGACCTGATCCA | | 75.97 |
| Ciz1-ex13 | TGCACCGTTTGCAACCGCTACTTCAAAACCCCTCGCAAGT | | 73.92 |
| Ciz1-ex14 | CTTGAGAAAGAAATTGCTGGCCAAGATGAGGACCACTTCA | | 70.85 |
| Ciz1-ex15 | ATATCCAGAGAGGAGTGGAAGGGCTCGGAGACCTACAGCC | | 75.97 |
| Ciz1-ex16 | TCTCCCACTGCAAGTCCCTGGGCCACTTTGAGAACCTGCA | | 75.97 |
| Ciz1-ex17 | GGCGCTCAACCCGCCTCAAAACCTGATAGAGGGACCTCCC | | 78.03 |
| Probes for less common predicted exons | | |  |
| Ciz1-ex1b1 | CGCGGGATGGCTGACCCTTCCGGGCGGCAGACGGACCTTG | | 82.12 |
| Ciz1-ex1b2 | CGAGCCTCCCCTTCGCGATGCGGAGGACGCGGGACAGAGG | | 82.12 |
| Ciz1-ex1c | TCCGTGGGGGCGACTTGAGCGTTGAGGGCGCGCGGGGAGG | | 83.15 |
| Ciz1-ex1d | GCGGAGTGGGGCGCGAGGAGGTGGCGGTGGTGGAGAGAAG | | 82.12 |
| Probes for most common predicted exon junctions | | |  |
| Ciz1-ex1a-ex2 | CGGCGGGAGCCGCAGAGCCACCATGTTCAGCCAGCAGCAG | | 81.1 |
| Ciz1-ex2-ex3 | CCCGTTGCCCATGGCTGTCAGCCGGGGGCTCCCCCCGCAG | | 84.18 |
| Ciz1-ex3-ex4 | CTTTGCTTTTACAGCAGTTGCAAGGACTGGACCAGTTTGC | | 71.88 |
| Ciz1-ex4-ex5 | CCACAGCAACACTGGGTAACCTCCGAGGCTATGGCATGGC | | 77 |
| Ciz1-ex5-ex6-a | CACCCCCAATCGAAAGGATTCTTCTTCTCAGACAATGCCT | | 71.88 |
| Ciz1-ex5-ex6-b | CCAATCGAAAGGATTCTTCTTCTCAGACAATGCCTGTGGA | | 70.85 |
| Ciz1-ex5-ex6-1 | ACCACCCCCAATCGAAAGGATTCTTCTTCTCAGACAATGC | | 71.88 |
| Ciz1-ex5-ex6-2 | TCCTCTACCACCCCCAATCGAAAGGATTCTTCTTCTCAGA | | 71.88 |
| Ciz1-ex 6-ex7 | TGGACACACCAGAAGACCAAGATTTACCGCCCTGCCCAGA | | 74.95 |
| Ciz1—ex7-ex8 | AGCTGCCAGCAAAGAGATTGAGGAGCTCAGAAGAGCCCAC | | 74.95 |
| Ciz1-ex8-ex9 | ATGCAGTAGAAGCTGGTGGAGGCATGGAAAAGACCTTGCC | | 73.92 |
| Ciz1-ex9-ex10 | AGAGCGAGAGAGATGCCAGGGGTATGGGGCGCCGGGGGCT | | 81.1 |
| Ciz1-ex10-ex11 | CTGCTCCAGCCAGCAGGAGTTCCAGGACCACATGTCGGAG | | 78.03 |
| Ciz1-ex11-ex12 | TGGAGACAGAGGATGAGGAGCCTCCACCAAGGCGCTGGTG | | 78.03 |
| Ciz1-ex12-ex13 | ACACCGCAGGACACAGGACCACAAGATTGCCAAACAATCC | | 73.92 |
| Ciz1-ex13-ex14 | GGGGCATAAGGACAAAGCCAAGGAGCTGAAGTCGCTTGAG | | 74.95 |
| Ciz1-ex14-ex15 | GGTTGAGGAGGAACTCTGCAAGCAGGTGAGGTCCAGAGAT | | 74.95 |
| Ciz1-ex15-ex16 | CAGCCCCAATACTGCATATGGTGTGGACTTCCTGGTGCCC | | 75.97 |
| Ciz1-ex16-ex17 | TTTGAGAACCTGCAGAAATACAAGGCGGCCAAGAACCCCA | | 72.9 |
| Probes for observed rare junctions | | |  |
| Ciz1-ex1b-ex2 | | ATGCGGAGGACGCGGGACAGAGGCCACCATGTTCAGCCAG | 79.05 |
| Ciz1-ex1c-ex2 | | GCGCGGGGAGGCGAGCCACCATGTTCAGCCAGCAGCAGCA | 81.1 |
| Ciz1-ex1ca1-ex5a1 | | CGCGGGCCGGAGCCGCCCCAGCCTCACACCCCCACAACTG | 84.18 |
| Ciz-ex1d-ex2 | | TCCCACGGAGCTCAGGACCACGCCACCATGTTCAGCCAGC | 79.05 |
| Ciz1-ex2a0-ex3a1 | | GCAGCTCCAGAGAGCTTTGCTTTTACAGCAGTTGCAAGGA | 72.9 |
| Ciz1-ex2a1-ex2a2 | | CAGCAACAGCAGCAGGCCCCGTTGCCCATGGCTGTCAGCC | 80.08 |
| Ciz1-ex2a3-ex5a1 | | AGCTCCAGCAGCTGCTCCAGCAGTCCCCAGCCTCACACCC | 80.08 |
| Ciz1-ex3-ex5 | | TGCTTTTACAGCAGTTGCAAGGTAACCTCCGAGGCTATGG | 72.9 |
| Ciz1ex3-ex5-1 | | AGAGAGCTTTGCTTTTACAGCAGTTGCAAGGTAACCTCCG | 71.88 |
| Ciz1ex3-ex5-2 | | AGCTTTGCTTTTACAGCAGTTGCAAGGTAACCTCCGAGGC | 72.9 |
| Ciz1ex3-ex5-3 | | TTACAGCAGTTGCAAGGTAACCTCCGAGGCTATGGCATGG | 73.92 |
| Ciz1ex3-ex5-4 | | AGTTGCAAGGTAACCTCCGAGGCTATGGCATGGCATCCCC | 75.97 |
| Ciz1-ex5-ex6a1 | | ACCACCCCCAATCGAAAGACAATGCCTGTGGAAGACAAGT | 72.9 |
| Ciz1-ex8a1-ex8a5 | | CTGCAGGTCCAGGCCCAGGTCCACACACAGGCACAGCCAA | 79.05 |
| Ciz1-ex8a2-ex8a7 | | AGGCAGAGCCACAGATGCAGCTGCAGCAGGAGGCAGAGCC | 79.05 |
| Ciz1-ex8a3-ex8a4 | | GCCACAGAAGCAGGTGCAGCCACAGGTCCACACACAGGCA | 78.03 |
| Ciz1-ex8a4-ex10a1 | | AGGCACAGCCAAGCGTCCAGCACTCCCTCTAAGCAGGCCC | 79.05 |
| Ciz1-ex8a0-ex12a1 | | AACCTCCAGGGCAGTTACAGGACACAGGACCACAAGATTG | 73.92 |
| Ciz1-ex8af1-ex13a1 | | GGGCAGTTACAGGTGAAGGCCCACCAGATTGCCAAACAAT | 73.92 |
| Ciz1-ex8af2-ex15 | | GCCCGGATGACAGTACCGAAACAGGTGAGGTCCAGAGATA | 74.95 |
| Ciz1-ex9-ex10-L_ins-a | | GAGAGAGATGCCAGGGTTCCTACTCCCCATGCAGGTATGG | 75.97 |
| Ciz1-ex9-ex10-L_ins-b | | AGATGCCAGGGTTCCTACTCCCCATGCAGGTATGGGGCGC | 78.3 |
| Ciz1-ex12-ex12+ | | AGGACACAGGACCACAAGCCACCCACACCACGAAGAGATG | 75.97 |
| Ciz1-ex12+-ex13 | | CACAGCCCGGCTTGTTACAGATATGATTGCCAAACAATCC | 71.88 |
| Ciz1-ex14a1-ex15 | | GATGAGGATGAAGAAGAGATCGAGGTGAGGTCCAGAGATA | 71.88 |
| Probes for hypothetical junctions | | |  |
| Ciz1-ex1ca1-ex2 | CGCGGGCCGGAGCCGCCACCATGTTCAGCCAGCAGCAGCA | | 82.12 |
| Ciz1-ex1c-ex3 | GGGAGGCGAGGGGGCTCCCCCCGCAGCAGCCACAGCAGCC | | 85.2 |
| Ciz1-ex1c-ex2a2 | CGGGGAGGCGAGAGGCCCCGTTGCCCATGGCTGTCAGCCG | | 83.15 |
| Ciz1-ex1c-ex4 | CGCGCGGGGAGGCGAGGACTGGACCAGTTTGCAATGCCAC | | 80.08 |
| Ciz1-ex1c-ex5 | GGGAGGCGAGGTAACCTCCGAGGCTATGGCATGGCATCCC | | 79.05 |
| Ciz1-ex1c-ex6 | GCGCGCGGGGAGGCGAGGATTCTTCTTCTCAGACAATGCC | | 78.03 |
| Ciz1-ex1c-ex6a1 | GGGAGGCGAGACAATGCCTGTGGAAGACAAGTCAGACCCC | | 77 |
| Ciz1-ex2-ex4 | GCCCCGTTGCCCATGGCTGTCAGCCGGACTGGACCAGTTT | | 79.05 |
| Ciz1-ex2-ex5 | GCCCCGTTGCCCATGGCTGTCAGCCGGTAACCTCCGAGGC | | 81.1 |
| Ciz1-ex2-ex6 | CCGTTGCCCATGGCTGTCAGCCGGATTCTTCTTCTCAGAC | | 75.97 |
| Ciz1-ex2-ex7-a | CCATGGCTGTCAGCCGACCAAGATTTACCGCCCTGCCCAG | | 78.03 |
| Ciz1-ex2-ex7-b | GCCCCGTTGCCCATGGCTGTCAGCCGACCAAGATTTACCG | | 78.03 |
| Ciz1-ex3-ex6 | GCTTTGCTTTTACAGCAGTTGCAAGGATTCTTCTTCTCAG | | 69.82 |
| Ciz1-ex3-ex6a1 | TGCTTTTACAGCAGTTGCAAGACAATGCCTGTGGAAGACA | | 70.85 |
| Ciz1-ex3-ex7 | TGCTTTTACAGCAGTTGCAAGACCAAGATTTACCGCCCTG | | 71.88 |
| Ciz1-ex4-ex6 | CCCACAGCAACACTGGGATTCTTCTTCTCAGACAATGCCT | | 72.9 |
| Ciz1-ex4-ex6a1 | GCAACACTGGACAATGCCTGTGGAAGACAAGTCAGACCCC | | 74.95 |
| Ciz1-ex4-ex7 | CCATGCCCACAGCAACACTGGACCAAGATTTACCGCCCTG | | 75.97 |
| Ciz1-ex4-ex8 | TCTCACCATGCCCACAGCAACACTGGCTCAGAAGAGCCCA | | 75.97 |
| Ciz1-ex5-ex7 | TCCTCTACCACCCCCAATCGAAAGACCAAGATTTACCGCC | | 73.92 |
| Ciz1-ex5-ex8 | TCCTCCTCTACCACCCCCAATCGAAAGCTCAGAAGAGCCC | | 75.97 |
| Ciz1-ex7-ex9 | GCCAGCAAAGAGATTGAGGAGGCATGGAAAAGACCTTGCC | | 73.92 |
| Ciz1-ex7-ex10 | CCAGCAAAGAGATTGAGGAGGTATGGGGCGCCGGGGGCTC | | 79.05 |
| Ciz1-ex9-ex11 | GAGCGAGAGAGATGCCAGGGGAGTTCCAGGACCACATGTC | | 77 |
| Ciz1-ex9-ex12 | ACAGAGCGAGAGAGATGCCAGGGGGAGCCTCCACCAAGGC | | 79.05 |
| Ciz1-ex10-ex12 | CTGCTCCAGCCAGCAGGGAGCCTCCACCAAGGCGCTGGTG | | 81.1 |
| Ciz1-ex10-ex13 | GCTGCTCCAGCCAGCAGATTGCCAAACAATCCTTGCGACC | | 75.97 |
| Ciz1-ex10-ex15 | AGCTGCTCCAGCCAGCAGGTGAGGTCCAGAGATATATCCA | | 74.95 |
| Ciz1-ex13-ex15 | GGCATAAGGACAAAGCCAAGGAGGTGAGGTCCAGAGATAT | | 72.9 |
| Probes for alternative exons containing intronic sequences | | |  |
| Ciz1-Intron 8-1 | TGGGACAGCTCTGCCCAGACCCTTCATTCCTCCCCTGACT | | 77 |
| Ciz1-Intron 8-2 | GGCAGCTCTTGGGCCTGGGGACTCTGGGAAATACAGCACA | | 77 |
| Ciz1-Intron 12 | CACGAAGAGATGTGTTTGCCCACGTTCCAGTGCAGGGGTG | | 75.97 |
| Ciz1-Intron 15 | GAAGCCTTCTCTTCTGCCCCTGCCTAGAGGGTTGGGGAGC | | 78.03 |
| Probes for other genes | | |  |
| ATM-ex22 | CGAAGGGAGATTCTTCCAGGTTACTGAAAGCACTTCCTTT | | 70.85 |
| ATM-ex41 | TGGGGCAAAGCCCTAGTAACATATGACCTCGAAACAGCAA | | 71.88 |
| ATM-ex61 | TTCACCCTACTCTGAATGCAGATGACCAAGAATGCAAACG | | 70.85 |
| ATR-ex11 | GTCAGATGACAGCACTTCCGAATACTCCATGCCAGAATGC | | 72.9 |
| ATR-ex19 | GTTGCCAAGATGCAAACTCTCAAGCTCGGTTGCTCTGTGG | | 73.92 |
| ATR-ex31 | TGCTCTCCTTAATGCAGGGGAATCACGACTCGCTGAACTG | | 73.92 |
| Cdc6-ex4 | CCAGCAAGCAAAGCTGGTCCTGAACACAGCTGTCCCAGAT | | 74.95 |
| Cdc6-ex6 | GGATGTATTGTACACGCTATTTGAATGGCCATGGCTAAGC | | 70.85 |
| Cdc6-ex11 | CAGCAGGTGGCGGCTGTGGACCAGTCAGAGTGTTTGTCAC | | 77 |
| cdc25-ex6 | CAGTAAGACCTGTATCTCGTGGCTGCCTGCACTCTCATGG | | 74.95 |
| cdc25-ex9 | TCCACCTGGAAGTACAAAGAGGAGGAAGAGCATGTCTGGG | | 73.92 |
| cdc25-ex15 | CCTGGGCAGGGGAGAAGAGCAAGAGGGAGATGTACAGTCG | | 77 |
| CDK2-ex1 | CCAAAAGGTGGAAAAGATCGGAGAGGGCACGTACGGAGTT | | 73.92 |
| CDK2-ex2 | ACTGAGGGTGTGCCCAGTACTGCCATCCGAGAGATCTCTC | | 75.97 |
| CDK2-ex6 | TGGGGACCCCAGATGAGGTGGTGTGGCCAGGAGTTACTTC | | 77 |
| CHEK1-ex5 | CGTGAGCGTTTGTTGAACAAGATGTGTGGTACTTTACCAT | | 69.82 |
| CHEK1-ex6 | CATGGGACCAACCCAGTGACAGCTGTCAGGAGTATTCTGA | | 73.92 |
| CHEK1-ex9 | TCTCAGCCAGAACCCCGCACAGGTCTTTCCTTATGGGATA | | 73.92 |
| CyclinE1-Ex5 | TCAGCACTTTCTTGAGCAACACCCTCTTCTGCAGCCAAAA | | 71.88 |
| CyclinE1-Ex7 | GCACCAGTTTGCGTATGTGACAGATGGAGCTTGTTCAGGA | | 72.9 |
| CyclinE1-Ex9 | GGATCTCTGTGTCCTGGATGTTGACTGCCTTGAATTTCCT | | 71.88 |
| CyclinE1-ex11 | AGGGCTTCTCCTCTCCCCAGTGGGCTCCTCACCCCGCCA | | 80.08 |
| CyclinA1-ex2 | TTGGAAAGCAAACAGTAAACAGCCTGCGTTCACCATTCAT | | 69.82 |
| CyclinA1-ex6 | TCAAGTATTTGCCATCAGTTATTGCTGGAGCTGCCTTTCA | | 69.82 |
| CyclinA1-ex7 | ATGGACCTTCACCAGACCTACCTCAAAGCACCACAGCATG | | 73.92 |
| MCM3-ex3 | CTGGTTGCCTTCCAGCGGGCCTTAAAGGATTTTGTGGCC | | 75.21 |
| MCM3-ex10 | GATCCTGAGCAGGATCGGGAGATCTCAGACCATGTCCTTC | | 74.95 |
| MCM3-ex17 | TGAAGGCATTCAAGGTGGCCCTCTTGGATGTGTTCCGGGA | | 74.95 |
| P21-ex2a | GACAGCGAGCAGCTGAGCCGCGACTGTGATGCGCTAATGG | | 78.03 |
| P21-ex2b | GGTCCCCAGGTGGACCTGGAGACTCTCAGGGTCGAAAACG | | 78.03 |
| P21-ex3 | TACCACTCCAAACGCCGGCTGATCTTCTCCAAGAGGAAGC | | 74.95 |
| P27-ex1a | CGTGCGAGTGTCTAACGGGAGCCCTAGCCTGGAGCGGATG | | 79.05 |
| P27-ex1b | GCCTTTAATTGGGGCTCCGGCTAACTCTGAGGACACGCAT | | 74.95 |
| P27-ex2 | ACAGAAGAAAATGTTTCAGACGGTTCCCCAAATGCCGGTT | | 70.85 |
| BRCA1-ex12 | TCATAAGTGACTCTTCTGCCCTTGAGGACCTGCGAAATCC | | 72.9 |
| BRCA1-ex15 | TCTGAAGACAGAGCCCCAGAGTCAGCTCGTGTTGGCAACA | | 74.95 |
| BRCA1-ex22 | CCACCCAATTGTGGTTGTGCAGCCAGATGCCTGGACAGAG | | 75.97 |
| CDKN1C-ex1a | CCTCGACGGCCTCGAGGAGGCGCCGGAGCAGCTGCCTAGT | | 82.12 |
| CDKN1C-ex1b | AGCCTCTCGCTGACCAGCTGCACTCGGGGATTTCGGGACG | | 79.05 |
| CDKN1C-ex2 | GCGCAAGAGATCAGCGCCTGAGAAGTCGTCGGGCGATGTC | | 78.03 |
| CDT1-ex4 | CCGGCCTGCCGGGACTCGTGCTGCCCTACAAGTACCAGGT | | 80.08 |
| CDT1-ex7 | CTTCAACGTGGATGAAGTACCCGACATCGAGCCGGCCGCG | | 78.03 |
| CDT1-ex9 | TGGCCTGTGCCAGGATGGTGGGCAGCTGTTGTACTATCAT | | 74.95 |
| CHEK2-ex2 | AGGACCAAGAACCTGAGGAGCCTACCCCTGCCCCCTGGGC | | 80.08 |
| CHEK2-ex4 | AAGGAAAACGCCGTCCTTTGAATAACAATTCTGAAATTGC | | 67.78 |
| CHEK2-ex9 | TTGACAAAGTGGTGGGGAATAAACGCCTGAAAGAAGCTAC | | 70.85 |
| DDX17-ex9 | TGTGGAGACAAAGAGACGCTGTGATGATCTGACTCGAAGG | | 72.9 |
| DDX17-ex12 | GTACCGCCTATACCTTCTTCACCCCAGGGAACCTAAAACA | | 72.9 |
| DDX17-ex13 | ATACACCTATGGTCAAGGCACCTATGGGGCAGCTGCTTAT | | 72.9 |
| DHX9-ex21 | ACACCAGAGATGTTCCGAACACCATTGCATGAAATTGCTC | | 70.85 |
| DHX9-ex23 | TCATCAATGAAGGAAAGCGGCTGGGCTATATCCATCGAAA | | 70.85 |
| DHX9-ex28 | TGGAGATGGTCCACGTCCTCCCAAGATGGCCCGATACGAC | | 77 |
| DYNLL1-ex2a | AGGACTCGGTGGAGTGCGCTACTCAGGCGCTGGAGAAATA | | 75.97 |
| DYNLL1-ex2c | AAAAATGCGGACATGTCGGAAGAGATGCAACAGGACTCGG | | 72.9 |
| DYNLL1-ex3 | CCACCTGGCATTGCATCGTGGGGAGGAACTTCGGTAGTTATG | | 75.45 |
| ERG-ex3 | CTGATGAATGCAGTGTGGCCAAAGGCGGGAAGATGGTGGG | | 75.97 |
| ERG-ex4 | CTCACCCCCAGCTACAACGCCGACATCCTTCTCTCACATC | | 75.97 |
| ERG-ex10 | GGGGGTATATACCCCAACACTAGGCTCCCCACCAGCCATA | | 75.97 |
| ETV1-ex7 | CCAGCTTTCTGAACCCTGTAACTCCTTTCCTCCTTTGCCG | | 73.92 |
| ETV1-ex10 | CCTGGACTGGTCGAGGCATGGAATTTAAACTGATTGAGCC | | 72.9 |
| ETV1-ex12 | GAAGCCCTTTTCTCCATGGCCTTTCCAGATAATCAGCGTC | | 72.9 |
| EWS-ex11a | AAAGCCCAAAGGCGATGCCACAGTGTCCTATGAAGACCCA | | 73.92 |
| EWS-ex11b | TCTCCCTTGCTCGGAAGAAGCCTCCAATGAACAGTATGCG | | 73 |
| EWS-ex11f | TGGCCCTGGGGGGCCCCCTGGACCTTTGATGGAACAGATG | | 79.05 |
| FEV-ex2 | CTTCAAGGACGGGAAGAACCCGAGCTGGGGGCCGCTGAGC | | 80.08 |
| FEV-ex3a | AGCGCTACGCCTACCGCTTCGACTTCCAGGGCCTGGCGCA | | 80.08 |
| FEV-ex3b | CCGCTGCCCTTCCCCGGCCTCTCCAAACTCAACCTCATGG | | 79.05 |
| FLI1-ex5 | GGCCTATAATACAACCTCCCACACCGACCAATCCTCACGA | | 73.92 |
| FLI1-ex7 | CCCCTTGGAGGGGCACAAACGATCAGTAAGAATACAGAGC | | 73.92 |
| FLI1-ex9 | CCACCAGCAGAAGGTGAACTTTGTCCCTCCCCATCCATCC | | 75.97 |
| LIMD1-ex3 | GAAAAGCCTTTTATTTTGTCAACGGCAAAGTGTTTTGTGA | | 66.75 |
| LIMD1-ex7 | GATGAGACCATCCGTGTCGTGTCCATGGACAGAGACTACC | | 74.95 |
| LIMD1-ex8 | TCAATGATGAAGATGGCCACCGCTGTTATCCGCTGGAGGA | | 73.92 |
| MATR3-ex12 | GATCAGAAGTTTGATCAAAAGCAAGAGCTTGGACGTGTGA | | 69.82 |
| MATR3-ex15 | TCTCCAAGTGATAAGAAATCCAAAACTGATGGTTCCCAGA | | 68.8 |
| MATR3-ex16 | CAGGTGCTGAATCTTCTGAGAACGCTGATGATCCCAACAA | | 71.88 |
| MCM2-ex8 | CTACGACCCCTCGCTGACTTTCTCTGAGAACGTGGACCTC | | 75.97 |
| MCM2-ex13 | GTGGGCAGCCACGTCAGACACCACCCCAGCAACAAGGAGG | | 79,05 |
| MCM2-ex14 | GCATCCCCATTACGGTGCGGCACATCGAGTCCATGATCCG | | 77 |
| PCNA-ex2 | CTGGGATATTAGCTCCAGCGGTGTAAACCTGCAGAGCATG | | 73.92 |
| PCNA-ex4 | ATGAAGATATCATTACACTAAGGGCCGAAGATAACGCGGA | | 69.82 |
| PCNA-ex5 | AGTAAAGATGCCTTCTGGTGAATTTGCACGTATATGCCGA | | 69.82 |
| RAD17-ex5 | AATGGGCCTTCTACATTAGAAAGCAGCAGATTTCCAGCGA | | 70.85 |
| RAD17-ex14 | CATCTTTAACAGAATTAGACTCACCTCGGTTGCCCTCTCA | | 70.85 |
| RAD17-ex19 | TGAACCCACTCAAGCCACTGTGCCGGAAACCTGGTCTCTT | | 74.95 |
| RB1-ex19 | GGACCCTTTTCCAGCACACCCTGCAGAATGAGTATGAACT | | 72.9 |
| RB1-ex22 | ACCTTGTCACCAATACCTCACATTCCTCGAAGCCCTTACA | | 71.88 |
| RB1-ex24 | GTGCTGAAGGAAGCAACCCTCCTAAACCACTGAAAAAACT | | 70.85 |
| TP53-ex6 | CTCAGCATCTTATCCGAGTGGAAGGAAATTTGCGTGTGGA | | 71.88 |
| TP53-ex7 | TACAACTACATGTGTAACAGTTCCTGCATGGGCGGCATGA | | 71.88 |
| TP53-ex10 | GCGTGAGCGCTTCGAGATGTTCCGAGAGCTGAATGAGGCC | | 77 |
| TP53BP1-ex26 | GAAGTACTTCCTGTGCCTTGCCAGTGGGATTCCTTGTGTG | | 73.92 |
| TP53BP1-ex27 | TCTGGTCTGAGATCCTCATGACTGGTGGTGCAGCCTCTGT | | 74.95 |
| TP53BP1-ex28 | AGTGGGTGATCCAGTGCCTCATTGTTGGGGAGAGAATTGG | | 73.92 |
| NP220-ex22 | TTGAATCCTTGTCCCAAGTGGGTCCAGTAAATGAGAATGT | | 69.82 |
| NP220-ex23 | CTCTGCCATCAGAAAAAGCTGTTGTGACAGAACCAGCAAA | | 70.85 |
| NP220-ex24 | GAAGACTCTTCTTCAGGCAAATCAGTGGCGTCTGATGTCC | | 72.9 |
| FGFR2-ex3 | ATCTCTCAACCAGAAGTGTACGTGGCTGCGCCAGGGGAGT | | 75.97 |
| FGFR2-ex4 | ACACCGATGGTGCGGAAGATTTTGTCAGTGAGAACAGTAA | | 70.85 |
| FGFR2-ex18 | TGGACCTCAGCCAACCTCTCGAACAGTATTCACCTAGTTA | | 71.88 |
| TNRC9-ex2a1 | GATTGTCACATCAGTCACCATTGCAGCCAACATGCCCTCG | | 73.92 |
| TNRC9-ex2a2 | CCCTCGAACATTGGGGCTCCACTGATAAGCTCCATGGGAA | | 74.95 |
| TNRC9-ex2b | GCCTTCTCCTCGGCAGCACTCCCCTGTCGCCTCTCAGATA | | 78.03 |
| VPS72-ex4 | GGAGGAACTGCTCCGGGAGGCCAAGATCACAGAAGAGCTT | | 75.97 |
| VPS72-ex5 | AGCGGCTCGAGGCTGATAAAAAGAAGCAGGTTCATAAGAA | | 70.85 |
| VPS72-ex6 | CAACCCCCCTGCTCGCTGCTCACGTACCTTCATCACTTTT | | 74.95 |
| Probes for control genes | | |  |
| AAMP-ex3 | CAATACCTTGGCAGTGACCGGGGGTGAAGATGACAAAGCC | | 74.95 |
| AAMP-ex8 | CAGCCCAGCCTGGGAGAAGGGGAGGAGAGTGAGTCCAACT | | 78.03 |
| AAMP-ex11 | TTGTGTCCAAAGGCCTGACCGTTAATGGCTGCAGCCCCTG | | 75.97 |
| Calnexin-ex6 | TTCCAAAATGGAATAGAATGTGGTGGTGCCTATGTGAAAC | | 68.8 |
| Calnexin-ex9 | CTGCTAAGATTCCAGATGAAGAGGCCACAAAACCCGAAGG | | 72.9 |
| Calnexin-ex12 | GCTGAAGAGCGCCCGTGGCTGTGGGTAGTCTATATTCTAA | | 73.92 |
| Histone3-ex1(53) | GCTTTAATTAAATAAATTGGATCAAAGGCCGTTCGAGGTA | | 66.75 |
| Histone3-ex1(116) | CAGAATTGGCATTTTGAGAGGTGATTGATACTGCTAACAA | | 67.78 |
| Histone3-ex1(264) | CCAGATTTGGGGAGGGGGTGATCGTGGCAGGAAAAGTTGT | | 74.95 |
| PTDSS1-ex5 | GGCATGGTCGTTTGCCGGTTTTTAGAGATGAGGACTTACC | | 72.9 |
| PTDSS1-ex6 | CAGTTCACTCCTGCTAGCTGGACCTATGTTCGATGGTTTG | | 72.9 |
| PTDSS1-ex10 | GCATGATTTGGTATGCAGAACACTATGGTCACCGAGAAAA | | 69.82 |
| ESD-ex7 | AAATGCCAATTTTCCAGTGGATCCCCAAAGGATGTCTATT | | 68.8 |
| ESD-ex8 | TTGCTCCAATTTGCAACCCTGTACTCTGTCCCTGGGGCAA | | 73.92 |
| ESD-ex9 | TCCAGGATCTCAGCTGGACATACTAATTGATCAAGGGAAA | | 69.82 |
| PTBP1-ex8 | CTCCGTATGCAGGAGCTGGTTTCCCTCCCACCTTTGCCAT | | 75.97 |
| PTBP1-ex13 | CTGTTTTCCAGCAATGGGGGCGTCGTCAAAGGATTCAAGT | | 72.9 |
| PTBP1-ex14 | AGGCCCTCATTGACCTGCACAACCACGACCTCGGGGAGAA | | 77 |
