# Supplementary figures and images for: Differential detection of alternatively spliced variants of Ciz1 in normal and cancer cells using a custom exon-junction microarray
Source: BMC Cancer. 2010 Sep 10;10:482. doi: 10.1186/1471-2407-10-482 (PMC2945943; doi:10.1186/1471-2407-10-482)

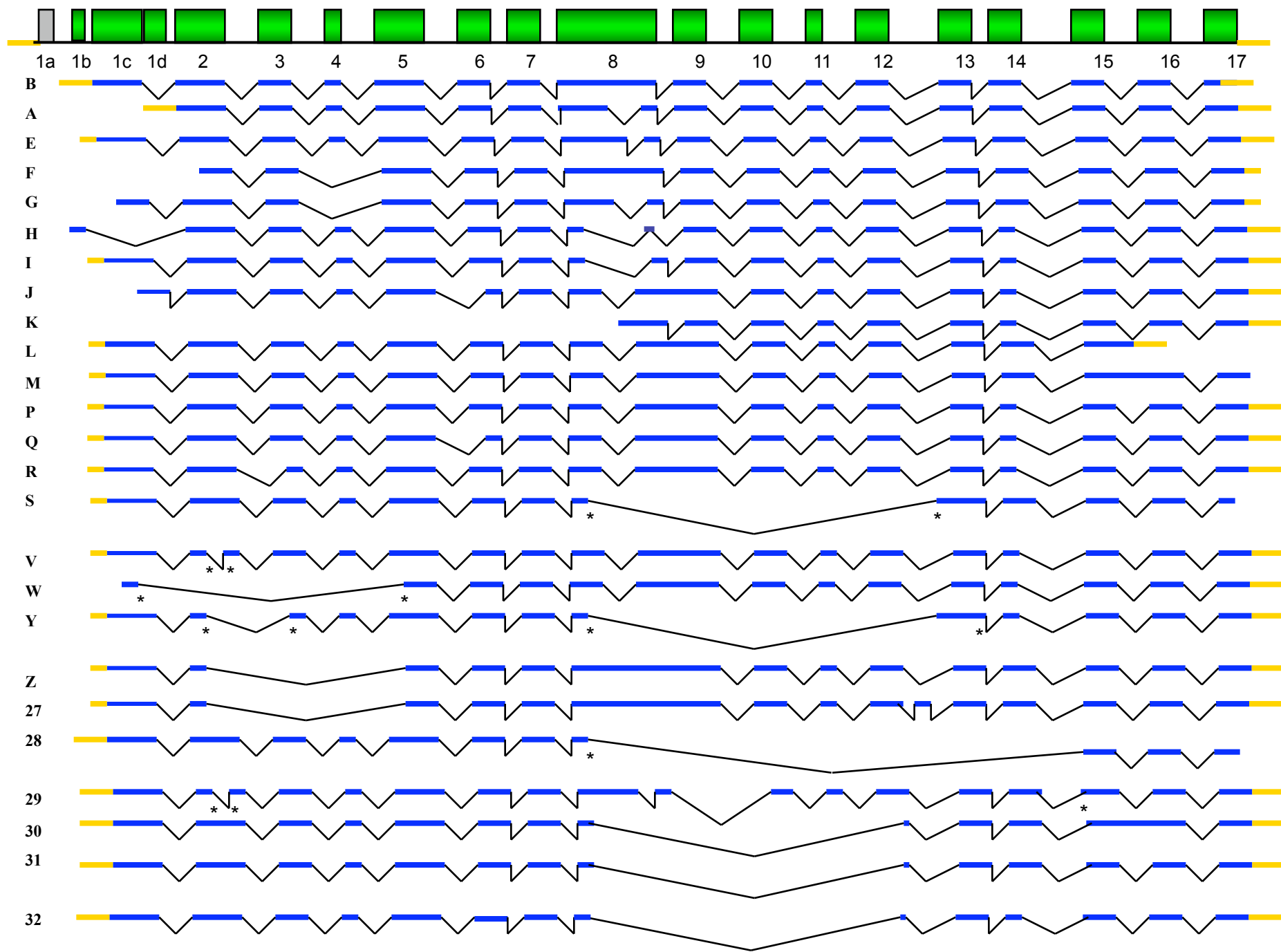

Supplement: Additional file 2 — Figure S1. Schematic representation of predicted human Ciz1 alternatively spliced transcripts assembled by AceView from 865 mRNA and ESTs submitted to GenBank [17]. Blue lines represent exons, black represent introns, yellow represent 5' or 3' untranslated regions and * represent non canonical exon-intron boundaries. Alternative transcript assemblies were designated, B, A, E, F, G, H, I, J, K, L, M, P, Q, R, S, V, W, Y, Z, 27, 28, 29, 30, 31 and 32 by AceView. [file 1471-2407-10-482-S2.PDF]

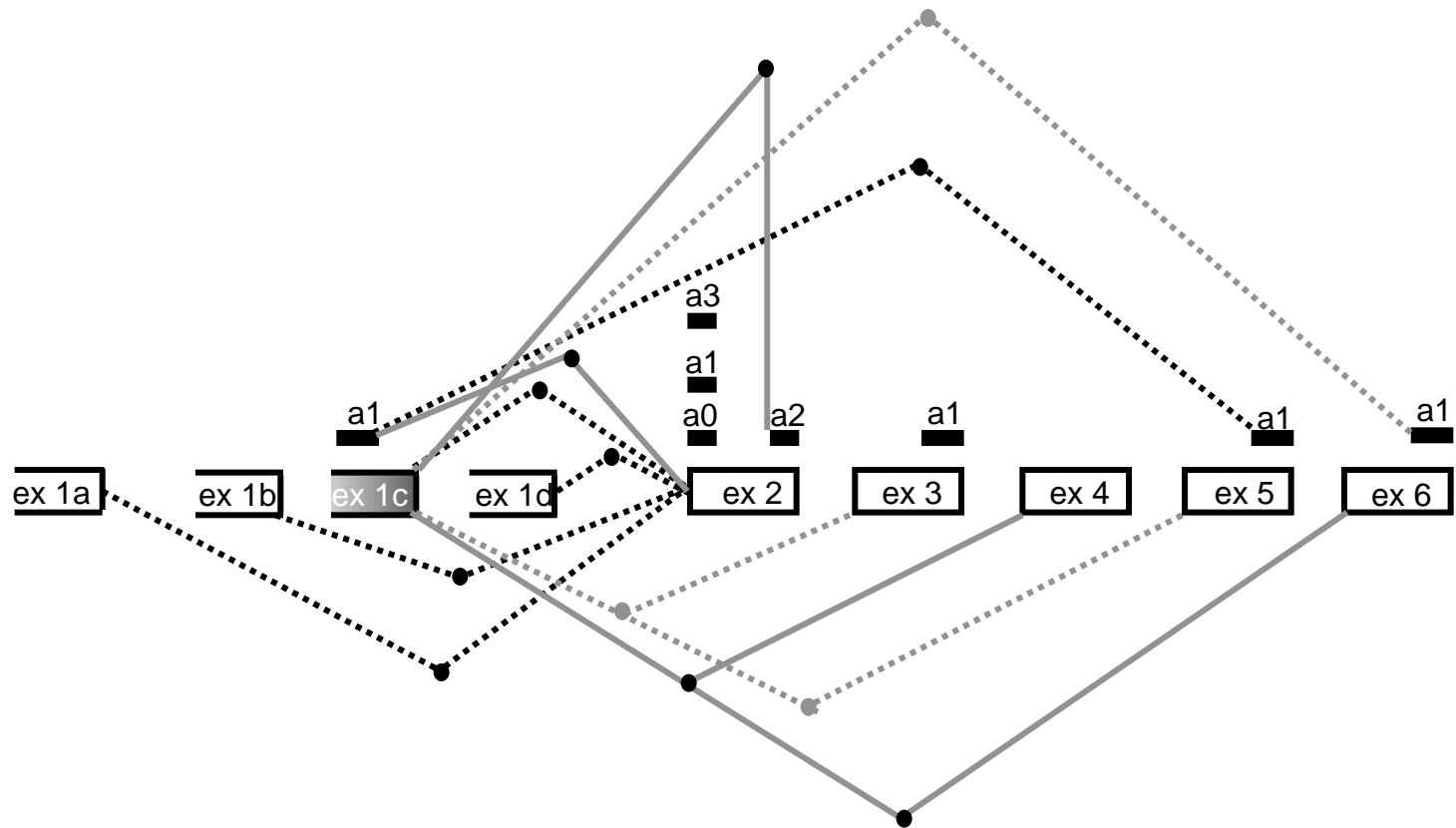

Supplement: Additional file 3 — Figure S2. Observed and hypothetical exon-junctions generated by alternative splicing of Ciz1 alternative exon 1 s (ex 1a, ex 1b, ex 1c and ex 1d). Common exons are indicated by labelled boxes and less common alternative exons by solid black boxes. Observed and hypothetical junctions are indicated by black and grey broken lines respectively. Splicing events that are over represented in TTC466 are indicated by solid lines. Sequences at 3' end of alternative exon 1 s are given in additional file 7 Table S14. [file 1471-2407-10-482-S3.PDF]
