# Supplementary material for: Differential detection of alternatively spliced variants of Ciz1 in normal and cancer cells using a custom exon-junction microarray
Source: BMC Cancer. 2010 Sep 10;10:482. doi: 10.1186/1471-2407-10-482 (PMC2945943; doi:10.1186/1471-2407-10-482)
Supplement: Additional file 4 — Table S2. Ranked product, top 4 up-regulated probes in technical replicates of SKNMC. Table S3 Ranked product, top 4 up-regulated probes in technical replicates of TTC466. [file 1471-2407-10-482-S4.DOC]

Table S2 Top 4 up-regulated probes (SKNMC)

| Probe name |
| --- |
| Ciz1-ex8a0-ex12a1 |
| Ciz1-ex12-ex13 |
| Ciz1-ex8af2-ex15 |
| Ciz1-ex1b2 |

Table S3 Top 4 up-regulated probes (TTC466)

| Probe name |
| --- |
| Ciz1-ex1ca2-ex2 |
| Ciz1-ex4-ex5 |
| Ciz1-ex1c-ex2a2 |
| Ciz1-ex1c-ex6 |
