# Supplementary material for: Differential detection of alternatively spliced variants of Ciz1 in normal and cancer cells using a custom exon-junction microarray
Source: BMC Cancer. 2010 Sep 10;10:482. doi: 10.1186/1471-2407-10-482 (PMC2945943; doi:10.1186/1471-2407-10-482)
Supplement: Additional file 7 — Table S14. Sequences of alternative exon 1 (3' end). [file 1471-2407-10-482-S7.DOC]

Table S14: Sequences of alternative exon 1 (3′ end)

| Alternative exon 1 Sequences at 3′ end |
| --- |
| 1b TCCCCTTCGCGATGCGGAGGACGCGGGACAGAGG |
| 1c CGACTTGAGCGTTGAGGGCGCGCGGGGAGGCGAG |
| 1d GGAGAGAAGCCGTCCCACGGAGCTCAGGACCACG |
